# Online Team-Based Learning Teaching Strategy for Developing Caring Competencies in Nursing Students under COVID-19 Pandemic Restrictions

**DOI:** 10.3390/healthcare9111510

**Published:** 2021-11-05

**Authors:** Tzu-Pei Yeh, Shan-Mei Chang, Ya-Fang Ho, Wei-Fen Ma

**Affiliations:** 1 School of Nursing, China Medical University, Taichung 406040, Taiwan; tzupeiyeh@mail.cmu.edu.tw (T.-P.Y.); smchang.nctu@cmu.edu.tw (S.-M.C.); avonho97@mail.cmu.edu.tw (Y.-F.H.); 2 Nursing Department, China Medical University Hospital, Taichung 404332, Taiwan; 3 PhD Program for Health Science and Industry, College of Health Care, China Medical University, Taichung 406404, Taiwan

**Keywords:** online team-based learning, teaching strategy, caring competencies, nursing students, COVID-19

## Abstract

(1) Background: The COVID-19 pandemic impacted education systems globally, and numerous strategies were used to transform education into online learning. Caring is recognized as a core competency in nursing; this competency is difficult to cultivate and measure. This study aimed to explore the effect of online team-based learning (TBL) on cultivating nursing students’ caring competency. (2) Method: A mix-methods study design with convenience sampling was used for this study. The intervention was online TBL with field observation. Quantitative data were collected by the modified Peer Caring Measurement (PCM) and analyzed using descriptive and inferential statistics. Qualitative data were collected by dialog in social media and analyzed by content analysis. (3) Result: Both the quantitative and qualitative data showed a significant increase in caring competency. A paired *t*-test of modified PCM showed significant improvement (*p* < 0.001), and female students had greater learning performances compared with male students in academic and affective dimensions. Three themes emerged, including that online TBL possesses remarkable benefits, students felt in charge of their learning, and changes in the students’ caring competency were revealed. (4) Conclusion: This online TBL strategy works well in teaching and fostering caring in an online environment among nursing students, which is necessary under COVID-19 restrictions.

## 1. Introduction

In nursing education, knowledge construction, skill acquisition, humanistic, ethical, and caring competencies are recognized as integral nursing abilities [1]. Caring is defined as a feeling of being loved and a kind of spiritual energy [2,3]; it can be revealed and perceived by mutual interaction in meaningful interaction through inner literacy with others [4]. Caring is the core value of nursing professionals, and delivering care is an essential ability in nursing practice [5]. It is crucial for nursing students to cultivate caring and empathy; these soft skills of caring and communication are not as high on students’ perceived needs as learning technical skills [6]. Training undergraduate students to have caring attitudes and use these skills has been embraced as one of the core competencies in all nursing fields [7,8].

Caring is considered an essential behavior to teach in nursing education; it can be taught, learned, and experienced [9] by understanding health needs or accumulating empathy. Numerous studies proposed that caring competencies need to be learned and applied in daily life, including care for self, peers, family, and even patients [10]. Teaching caring in a practical and effective approach in the classroom and clinical settings remains a challenging task, especially in a traditional teacher-centered course design. However, the teaching efficacy in class or online has been diminished because of the coronavirus restrictions in 2019, which posed tremendous challenges for humanity [11]. This negative impact involved the 2021 education system in Taiwan [12,13] and 2020–2021 worldwide [14,15]. How to deliver knowledge, attitude, and skills of caring through an online course is a tremendous challenge.

A collaborative learning–teaching approach was first developed by Larry Michaelsen in 1987 [16]. Team-based learning (TBL) emphasizes students’ self-learning and preparation out of class and in-class and application of knowledge through small-group discussions (also known as “application activities” or “modules”). Different from other group-oriented learning methods, TBL uses more group discussions, group presentations, or group assignments in a class; meanwhile, the individual students still represent themselves. Instead, TBL emphasizes students working as a team, in which all members complete activities systematically and strategically together under a well-organized course design. This approach will be taken as the backbone of the entire class. In-class discussions require students to put knowledge and skills they have learned from class into actual applications in real situations [17]. The inter- and intra-group discussions stimulate interactions and dynamics and may help expand both the vividness and depth of teaching [18], and the benefit is consolidated by students’ being well-preparing before every lesson by previewing online lectures. In a study that made participants experience several diverse teaching approaches, TBL was voted by the majority of participants as their favorite method. This result indicated that most students like learning through TBL [19].

In recent decades, TBL has been extensively applied in medical and nursing education worldwide, including America, Japan, Korea, and Singapore, especially in courses related to basic medical science, clinical medicine, nursing, medical laboratory science and biotechnology, and pharmacy [20,21]. In a study that reviewed 118 research articles between 2011 to 2016 [22], 61% of students who participated in TBL were students with majors that were medically related. It is clear that TBL is compatible with the needs of medical fields’ teaching and learning. This design helps students cultivate a responsible learning attitude and enhance their abilities in independent learning and in tackling the challenge of handling teaching materials [23,24,25,26].

Cheng et al. [27] indicated that TBL significantly improved students’ learning autonomy and in-class participation and promoted teamwork skills and overall performance. In a review article by River and colleagues [28], nine studies with a total sample of 2094 students enrolled in nursing courses that adopted TBL were included. Most instructors incorporate blended learning into their TBL classrooms by using e-learning and online materials to boost students’ interest and engagement. Another study assessed 60 nursing students who took a TBL course; the results revealed better performances in teams of stronger group cooperation [29]. Therefore, the bonds and links among team members were the key factors of successful TBL.

The purpose of this project was to increase students’ sensitivity and competencies in caring for themselves, their families, peers, community, and the environment prior to actually working as medical professionals. Furthermore, this course aimed to enhance students’ sensitivity to others’ health needs, which is an important component of caring competence, by using the TBL teaching strategy with field observation.

## 2. Materials and Methods

### 2.1. Study Design

This research applied a mixed-methods research design. Quantitative surveys were completed as pre-test and post-test for measuring the changes in students’ caring competency. All the interactions on the social media platform in Line were downloaded as a text file for further qualitative analysis.

### 2.2. Research Participants

This study recruited all first-year nursing students enrolled in the course “Nursing and Life” during the COVID-19 pandemic. This course aimed at cultivating caring competencies of college freshmen; the course was offered by the School of Nursing of a medical university in Taiwan. A total of 103 students participated in this course, and 97 of them completed the study.

### 2.3. Study Intervention

**Figure 1:**
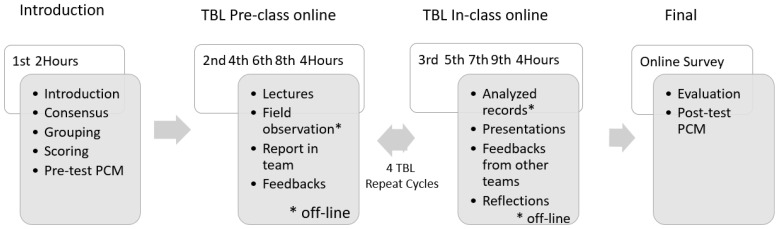
The process of conducting “Nursing and Life” online TBL course (9 weeks biweekly). TBL: online team-based learning; PCM: peer caring measurement. *, offline learning.

The interventions in this study included online TBL teaching, field-study-styled social observation records of students, pre-class online lectures, and discussions in Line (commonly used in Asia). All interventions were conducted in the “Nursing and Life” course. Each lecture was conducted for 4 h biweekly within 9 weeks. Except for the first lecture, four team-based presentations and discussions were run, and each TBL took 4 h. In the first week, the course introduction, grouping in teams, and scoring methods were displayed to students and reached consensus by students and teachers. The course evaluation and post-test of PCM were arranged after the final lecture with online survey. The process of this online TBL course is demonstrated in Figure 1.

During TBL sessions, the TBL pre-class online lectures with stories were used to initiate students’ learning motivation. Four stories included culture sensitivity, career planning, nursing profession, and social care and self-help in vulnerable populations. After watching the online lectures, the field-study-style social observation record sheet designed by the instructor was used to guide students to observe people and surrounding environments in selected places outside the classroom. The observation records in different locations were completed as the individual team assignments. The observations mainly focused on exploring the observed people’s health needs. The observation locations could be selected by students, but each observation had its limitation, for example, outside the classroom, and students must not know the observed person. Interaction except inevitable conversation should be avoided between students and observed targets. In addition, the students were asked to avoid the observed people’s awareness of being observed.

Each team was composed of two to three students. On returning from their observation, students discussed their experiences in the online discussion room in Line. Next, students were assigned learning tasks of analyzing the observation records according to the guidance offered by the instructors based on essential TBL concepts and strategies. The observation records should include dates, start time, place, environment description, the outlook, characteristics, and behaviors (including interactions with other people) of the observed people, the possible thoughts or emotional status of the people, and finally, judgments on the potential needs with reasons predicted by students. Students were requested to complete the observations in as much detail as they could and complete the tasks without interaction with target subjects and only once for each observation.

After this work, students were given a chance to learn from other teams’ observation reports through class-wide discussion and mutual feedback to motivate them into re-examining the pros and cons of their own perspectives and viewpoints. Line, a commonly used social media in Asia, was used for reporting, sharing, and answering questions, group discussion, and even briefly presenting the observation results instantly. The TBL strategies lead the course conduction, students’ learning, and were used by teachers to stimulate the students to express various points of view; teachers aimed to cultivate students’ sensitivity to the public and to have empathy for people’s needs, especially vulnerable people. The group discussion and assignments make students brainstorm and learn from others’ feedback; students were expected to understand others’ needs, perceived self-feelings, have ability to reflect, accumulate caring competency, and practice using caring skills throughout the course. Thereby, this activity may train them to have critical thinking and develop their sensibility towards the needs of the people around them, even the surrounding environment and communities. Teachers played important roles in leading discussions and pointed out key elements or points of view in caring competency from students’ speech.

### 2.4. Instruments for Data Collection

Quantitative data were collected in demographic survey and modified Peer Caring Measurement (PCM), and the qualitative data was collected from social media—Line records. After each field-study-style participant social observation activity, students presented their observation results and had group discussions in online TBL via Microsoft Teams (Microsoft Corporation, Redmond, Washington, US); due to the time limit, feedbacks were given via Line (social media) group. The conversations, interactions, feedback, and reflections in Line were downloaded as a text file for further qualitative analysis.

#### 2.4.1. Personal Data

The questionnaire begins with a demographic survey with 10 items for personal information, including age, gender, major, birth order, height, weight (for calculating body mass index (BMI)), self-reported family financial status, with or without student loan, reliance on family financial support, and compatibility of current major and personal interest.

#### 2.4.2. The Modified Peer Caring Measurement

The Peer Caring Measurement (PCM) was developed by Kuo and colleagues [30] to measure nursing students’ peer caring behaviors. The PCM is a Taiwanese culturally sensitive instrument that was developed to measure peer caring behavior from the student perspective in Taiwan. The PCM includes 17 items with 5-point Likert scale ranging from 1 (strongly disagree) to 5 (strongly agree). The 17-item PCM was reported to possess good internal consistency reliability with Cronbach’s of 0.9347 and with Pearson’s correlation coefficient of 0.72 in test–retest stability reliability among 47 nursing students [30]. The PCM has three dimensions, including nine items on assistant caring competency, four items on academic caring competency, and four items on affective caring competency. These three dimensions may explain a total of 63.197% of the variance in caring competency among 360 nursing students [30]. In this study, to match the course goal, the researchers added three extra items to measure the social sensitivity dimension for vulnerable populations. The modified PCM has 20 items, including three new items: “I will pay attention to the issues of the socially vulnerable population”, “I can empathize with the needs of socially disadvantaged people”, and “I can identify the information of the socially vulnerable population or disadvantaged groups”. The Cronbach’s alpha of the 20 items was 0.937 in 97 students of this study.

#### 2.4.3. The Conversation Text in Social Media

Students reported team-based field-study-style participant social observations briefly by points in Line group after each activity. A group presentation of field-study-style participants’ social observation results was held via Microsoft teams. The presentation time was tight so that the feedbacks and reflections on the presentations were proposed in the Line group simultaneously. Group interactions, dynamics, teachers’ feedbacks and guiding discussions, students’ changes in caring competency, and presentation skills were also recorded in the Line group conversation. Later, those conversations were downloaded as text for further analysis.

### 2.5. Data Collection and Ethical Consideration

The course design of “Nursing and Life” with online TBL obtained consensus among instructors. The method of conducting the course was revealed to students; the pre-test of modified PCM and grouping in teams were completed via online lecture in the course introduction in the first week. The four observation records and online presentations were conducted in teams. At the end, the post-test of modified PCM and course evaluation was completed online. The exemption from informed consent for this study was approved by the institutional review board because the research involved no more than minimal risk to study participants. Verbal consent was obtained from students. The researchers provided a brief explanation of the study purpose and a clear and concise study flow chart to students in class. Students’ privacy was protected, and they were free to accept or decline the survey. Any of their rights in the course would not be influenced whether they participated in the study or not. All the completed questionnaires remained anonymous, but a serial number on the dataset was marked. Connection between these code numbers and personal information was kept confidential.

Ethical review and approval were waived for this study due to it meeting the criteria of the Ministry of Education, Taiwan of exemption in obtaining Institutional Review Board approval of conducting the Teaching Practice Research Program. This study did not involve extra interventions in different groups and any specific changes in the course. Students’ privacy was protected, and they were free to accept or decline the survey. Any of their rights in the course would not be influenced whether they replied the survey or not. All of the completed questionnaires remained anonymous, but a serial number on the dataset was marked. The connection between these code numbers and personal information was confidential.

### 2.6. Data Analysis

Data analysis contained two parts in this study. The first part focused on the pre-and post-test quantitative data by using SPSS (Statistical Product and Service Solutions) version 22.0 (IBM, Armonk, NY, USA). The data were analyzed by descriptive statistical analysis, independent *t*-test, and paired *t*-test.

The second part centered on the text of the group presentations, discussions, sharing reflections, and feedbacks; the data were analyzed using content analysis. Content analysis is widely used by researchers who focus more on the data itself rather than giving an interpretation; also, this method is flexible for analyzing all kinds of data [31]. Content analysis was used for qualitative descriptive data in order to explore if this TBL course could cultivate students’ caring ability. The data were entered in NVivo version 11 (QSR International, Doncaster, Australia) to go through the data repeatedly to find emerging patterns.

The steps of content analysis in this study included open coding from reading through the data and searching for meaningful words, sentences, or paragraphs; allocating meaningful sentences under nodes with given names; and categorizing the nodes into themes. Then, all themes were given identifications and explanations for full understandings of what students had learned in this course. During the above, two researchers who were involved in this course and had experience in conducting qualitative studies checked the analysis and themes independently to ensure consistency and agreement in coding [32]. After coding, the researchers read the codes and themes again to remove inadequate codes by referring back to the raw data. Analogous themes were merged when all researchers agreed. The study followed the four criteria proposed by Lincoln in 1985 [33] to refine the concept of trustworthiness—credibility, transferability, dependability, and confirmability. A participating student was invited to check the themes and interpretations.

## 3. Results

### 3.1. Demographic Information

A total of 97 students participated in this course: 74 were female and 23 were male. The mean age was 20.15 (SD = 5.58), ranging from 18 to 47 years old. The total scores for caring competency and sub-scale scores showed normal distributions (Table 1). The mean scores of total caring competency at pre-test were 103.84 (SD = 10.29) and 108.75 (SD = 9.25) in post-test. The paired *t*-test between pre- and post-tests of caring total scale was 6.841 (*p* < 0.001, 95% C.I. (confidence interval) = 4.181–7.614). These results show that the caring competency was improved by the online TBL strategy in undergraduates in this study.

### 3.2. Gender Differences in Caring Competency Improvement

The independent *t*-test analysis showed that no gender differences were found in the pre-test (*t* = 0.269, *p* = 788) or post-test (*t* = 0.075, *p* = 0.940) of the modified PCM. However, female students had significantly better learning performances in cultivating caring competency than male students in academic and affective dimensions (Table 2). Female students showed significant improvements in total modified PCM scores and all sub-scales from pre-test to post-test. In male students, the two sub-scales of assistance and social sensitivity in caring competencies had significant improvements from pre-test to post-test. The overall modified PCM score at post-test was significantly higher than pre-test. Therefore, the results showed the online TBL strategy had positive improvements in total caring competency for both female and male students.

### 3.3. Content Analysis of Online Text in Social Media

Content analysis was used to analyze the group discussion in Line to illustrate the advantage of online TBL learning, students’ reflection, and their promotion of the caring competency as revealed in the group discussion. Three main themes emerged from the content analysis.

#### 3.3.1. Theme 1—The Online TBL Teaching Method Possesses Remarkable Benefits to Both Students and Teachers

##### The Online Course Offered Flexibility and Advantages for Interactive Responses between Students and Teachers

The teachers were no longer limited in their teaching in terms of locations and time. The online course processes and various tasks were explained clearly and directly and could be answered immediately if students had any questions.

*“Here are some reminders from teachers, the first time community field-study-style social observation today should include: (1) time, (2) location/place, (3) target subject’s characteristics, and (4) what you think about his/her needs.”*

(Line 560–564.)

The efficacy of teaching and learning were both promoted by students who could ask questions and obtain feedback or clarifications from teachers immediately. When the group discussions or oral presentations were ongoing, students could ask questions whenever they needed; the presenting students had opportunities to answer the question, and the teachers could give feedback and offer further clarification. This helped the students to concentrate even though all of the conversations were online.

*One student proposed the observation scenario of the target subject: “he looked very hungry but still using his smartphone … like he already has been hungry for three days”; teachers and other classmates asked this student: “how did you know he might be very hungry from your observation? Why three days? How did you know?” The student responded: “Because his long face looked like my face when I was hungry for three days!”*

(Line 1063–1213.)

##### The Online Approach Resolved the Problem of the Need to Avoid Physical-Attendance Courses during the COVID-19 Pandemic

Due to the coronavirus restrictions, teachers and students were prohibited from entering the university campus; all courses needed to be changed to online. Therefore, online TBL replaced the TBL approach, and crowded gatherings were avoided.

*“The online course kept the same units and contexts as the original design, as well as the TBL model; but all teachers and students will use ZOOM to participate in lessons instead of in-class lessons”*

(Line 424–425).

The students expressed that this online course was the first one that involved whole-time participation with many interactions, and all teachers and classmates joined the group presentations and discussions simultaneously. Those strategies made students feel involved and closely connected.

*“This is the first time that I paid attention and listened to the contexts from beginning to end in an online course.”*

(Line 2113.)

##### This Online TBL Course Design Promoted Students’ Positive Studying Attitude

The online TBL allowed students to give feedback to others and reflect on themselves in terms of positive learning, self-affirmation, increasing self-expectation, and diligent presentations. In addition, online TBL expanded the students’ opportunities in multidimensional learning. From team presentations, in terms of the needs of observed subjects, teachers and other team students may propose various needs under the same scenarios, which were different from the presenting team.

*“I like this presentation so much, and I appreciate other classmates’ well-planned presentations. I feel like everyone prepared their presentation full-heartedly; they really planned their lives in the next ten years seriously. When they presented with great enthusiasm, I also became hopeful for my future life.”*

(Line 2790–2794.)

*“You mentioned the subject looked twitchy, was it possible that the subject did not feel bored but was in a hurry?”* (Line 1450–1451); *“Had you considered the need of a stress-resistant?”* (Line 1452); *“Had you considered that the subject had low blood sugar?”* (Line 1457–1458); *“Maybe you may add the needs of relieving tiredness or easing of stress?”*

(Line 1460–1461.)

#### 3.3.2. Theme 2—Students Were in Charge of Their Own Learning Process

##### The Learning Attitude Was Changed into a Proactive Style

When students were sharing thoughts in team presentations, they expressed being inspirited by others’ presentations and willing to learn actively.

*“Each team had a brilliant presentation! From diversely observed dimensions, I found that there were many objects or needs that I had never thought of before. This made me assimilate more and realize that I still have to learn in many ways.”*

(Line 2210–2213.)

##### Promoting Abilities in Self-Reflection and Critical Thinking from Multi-Dimensions

The classmates expressed that they had expanded their own insights from others’ viewpoints in team presentations; this represented the processes of self-learning and self-reflection. In addition, the stimulation from teachers and classmates made the learning effects more in-depth, and learning dimensions were expanded.

*“I think Wang has her own specific reasons for her goals and motivations, and she has detailed plans and thoughts of her future. There are many valid thoughts and considerations which are worthy of learning; I hope I can be her friend for life even after graduating from the university.”*

(Line 2753–2756.)

*“I’m very impressed by the team 3 presentation, which portrayed behaviors of a middle-aged man who was buying cigarettes. I may connect that scenario with my own life, because I usually stamp my feet when I am anxious. Then, the presentation in team 2 about beef noodles is great, too.”*

(Line 2181–2184.)

##### The Inner Power of Learning Transformed into Lifelong Learning

The online TBL impacted students through their inner desires for learning, and the inner power of learning was activated and performed as lifelong learning mechanisms. The course goal of cultivating the caring competency may be maintained for long-term effects.

*“Teacher: Although this course emphasizes the affection of learning rather than knowledge and skills, we still expect that students may understand themselves, have better perceptions of their future life directions, realize the nursing profession in different dimensions, increase their sensitivity to their surroundings, and reinforce intrinsic power; so that you can make the most helpful choices for yourself, to others and the public, in any period of your lives.”*

(Line 2925–2930.)

#### 3.3.3. Theme 3—The Metamorphosis of Students’ Caring Competency Was Seen from Their Observations and Changes in View

##### The Observation Insights Focused on Individual Subject Were Changed from Simple to Complex Scenarios

The students changed their focus and thinking from physical needs to psychological needs, from needs in a single dimension to combined dimensions; furthermore, their sensitivities to the whole scenarios and the subjects’ emotions were increased gradually.

*“The characteristics of the subject: a male, wearing a glass, in trousers and a black T-shirt. The subject’s needs: (1) seems to be thirsty, needs water, (2) feels very hot because of the black T-shirt, needs to move into a cooler place, (3) the urge to smoke, agitated, need smoking, (4) chatting for a long time, may feel aches and sore feet, needs to seated.”*

(Line 737–739.)

The descriptions of observations became more and more detailed; the scenarios became more and more complex. The observed phenomena included physical reactions, interactions between the subject and other people, emotions, and atmosphere.

*“ (1) place: Chaotian Temple, (2) subject’s characteristics: wrinkles on the face, wearing a light jacket and comfortable leisure shoes, a hat from Chaotian Temple, walking toward the temple, mumbling, praying to Buddha devoutly, (3) what is the subject possibly doing at the moment: igniting an incense stick for prayer, (4) what are the possible needs of the subject: finding someone to listen to her worries, trust from God, a protective talisman from God for herself or for her children.”*

(Line 1524–1530.)

##### The Perceptions on Oneself Were Turned into Other People’s Feelings, and Empathy Was Aroused

Students were inspired in learning and having empathy. They learned from multi-dimension insights and felt empathy. Meanwhile, students felt empowered and energized from helping others; after they reflected on what they had experienced, they thought that they were more willing to help others and were more capable of being helpers.

*“This course made me know how to observe the changes of other’s facial expressions or actions; this may help me to predict what they are thinking so I can avoid offending them. I think this is quite helpful in clinical in the future; when I see anything wrong in a patient’s face or action, I might know they have special needs immediately. Helping others makes me have more energy, greater energy makes me help more people!”*

(Line 2251–2254.)

##### Approval of Themselves and Peers Increased Their Acceptance of Adverse Events

Students approved their own and peers’ devotion to positive learning by sharing their own experiences and thoughts. Their self-affirmation and self-expectation were increased. This is the ability to care for themselves and their peers.

*“My family stands against me majoring in nursing, and their reasons are just like most people think: nursing is tough work and unskilled work. Nurses take the risk of being attacked by patients, and they are also influenced by physical, mental and spiritual health due to frustration. However, my future belongs to me, and no one can decide for me. I really appreciate that my family changed its position and supported me! My family’s support is the most important motivation for me to be a nurse, especially because of the news of an isolated COVID-19 patient attacking and killing a nurse with a knife. I think most people feel fear of being a nurse after watching this news, and this is the reason why parents are opposed to supporting their children’s study of nursing. But if we think carefully, why do nurses still insist on their jobs even though many unfair cases happened? We may imagine that if every nurse fears nursing jobs, then the number of nurses will keep decreasing, and the quality of care for patients will be poor and poor. In my opinion, nurses not merely help society, but also help their loves.”*

(Line 2449–2463.)

## 4. Discussion

This study applied TBL online methods and aimed to cultivate first-year students’ caring competency; the evaluation of the performances included quantitative and qualitative methods. The results indicated that this course with online TBL design successfully promoted the first-year students’ sensitivity and behaviors in caring. The top five improved performances in caring were: concern about the vulnerable issues in society, being empathetic about the needs of vulnerable people, identifying the vulnerable population, encouraging other people, and being inspired. The teaching strategies used in this study indicated the efficacy of cultivating caring competency, especially in the sensitivity to others’ health needs.

### 4.1. The Cultivation of Students’ Caring Competency

From the students’ performance in the quantitative survey before and after class, accompanied by the qualitative presentations, the students showed high willingness to participate in online activities, the abilities of reflection and critical thinking, in-depth learning with diverse learning methods, and the learning process of caring. McMahon and Christopher [34] pointed out the cultivation of caring in nursing can, through observation and learning to anticipate the patient’s needs, develop reflective and critical thinking skills in nursing to identify patient’s vulnerability and threats. Empathy is key to the caring process and is characterized by trenchant and intuitive insight into another’s suffering [35]. Therefore, caring consists of an innermost core that attention to the patient’s suffering and needs wants to help [36]. Just like our study, the cultivation of the caring competency may be recognized in: (1) the increased precision of observation, (2) increased sensitivity to people’s needs, (3) increased empathy, and (4) increasing willingness to help people.

The increasing precision of observation could be shown from the students’ observation reports, including the needs of target participants changed from physical needs to psychological needs, the change in scenario descriptions from simple to complex, the observation participants from a single person’s behavior to interactions in a group of people. The increasing sensitivity to people’s needs could be confirmed because the needs changed from simply including the physical dimension to extension into psychological needs. Empathy was increased through the students’ pondering, inspiration, and feelings of empathy. Through participating in the course, the students were enlightened on their inner energy and obtained positive recognition of themselves and other people; this increased their willingness to help people.

### 4.2. The Advantage of Online TBL

TBL is a student-centered and self-motivated learning method; teachers may obtain immediate feedback from the online interactions with students [27,37]. The course contents, progression speed, and even evaluating requirements could be very flexible and meet the students’ expectations. Teachers’ timely appreciation of students’ positive performances made students more spontaneous in learning in a virtuous cycle [38]. Teachers’ guidance may induce students to have deeper thinking and point out the various thoughts from different peers who possess a variety of social–economic statuses and had various thoughts on the same scenarios. In addition, caring characteristics represented in conversations were emphasized by teachers to enforce the concept of caring.

TBL possesses both the advantages of in-class lecture and problem-based learning (PBL) [39,40,41]. In terms of teaching benefits, one instructor may lead several teams in a group discussion simultaneously; this could not only reduce the teacher’s manpower needs and time consumption but also avoid the teacher’s tiredness of repeating the same course content [42]. TBL is gradually and broadly used in many professions; it keeps large student numbers for in-class teaching but has the advantages of PBL, which requires numerous teachers and equipment [40,43,44].

However, TBL’s implication has difficulties in Taiwan. Taiwanese students are trained under a grades-priority culture; those students who have better performance in paper examinations would worry that TBL is not sufficient for dealing with various national exams [45,46]. This course is an optional course; the researchers (teachers) must be cautious on the time pressure for learning [19], which in this study meant the time pressure of team responses in online group presentation and discussion.

The greatest limitation to TBL efficacy depends on how much the teachers understand the implied meanings and spirit when applying TBL; besides, teachers have to design topics and related questions for each lesson according to the course goals [47]. The four rules of question design are the 4Ss [47,48]: (1) significant problem—important issues or scenarios which match the real field area, (2) same question—all teams discuss the same topic at the same time, (3) specific choice—each team has to decide on a specific answer to the question, and (4) simultaneous report—all teams answer simultaneously. This study not only fitted with the 4S rules by using community observation and online teaching, but this course design also revealed its flexibility and meanings under COVID-19 restrictions with various challenges. In addition, students’ observation reports may be kept on schedule; therefore, this online TBL is more concise in time control.

### 4.3. Limitations

Any online teaching method requires information technology equipment such as computers, tablet computers, and also good internet speed. Teachers must be familiar with operating online teaching software such as Google Meet, Microsoft Teams, Webex, etc.; and well-designed in-class activities, influent teaching process, and appropriate course materials have to be prepared in advance. The study performance is hard to evaluate, particularly via online; therefore, the blended teaching method and multi-dimension evaluations should be used, and all the rules and regulations need to be revealed to students clearly at the beginning of the course. Caring competency is a literacy that involves knowledge, attitude, skill, and application; in future studies, more reliable and valid measurement tools are needed. In addition, this course could be offered to other medical or health-care-related major students, even in junior high school, to cultivating caring as a core competency.

## 5. Conclusions

This online TBL “Caring in Practice” course successfully improved overall teaching efficacy under the COVID-19 pandemic and deepened the students’ learning experiences by real-life practice with observation and emulation. This study showed that online courses with well-designed and pre-class preparation may still accumulate students’ caring awareness, attitude, and skills by resolving real-world health problems. TBL has been approved such that it may remain work as an online course; it is important to know that the learning efficacy, students’ performance, and student preference all support that online TBL is a valid choice in university education. In this difficult time of COVID-19 restrictions, online TBL shows it is safe and effective in achieving the cultivation of nursing students’ caring competency in university education.

## Figures and Tables

**Table 1:** The modified PCM scores report at pre-test and post-test.

Dimension	Pre-Test				Mean (SD)		*t*	*p*
	Minimum	Maximum	Skewness	Kurtosis	Pre-Test	Post-Test		
Assistance	35	54	−0.488	−0.382	47.45 (4.71)	49.38 (3.91)	5.62	<0.001
Academic	16	24	−0.161	−0.734	20.77 (2.31)	21.55 (2.30)	4.36	<0.001
Affective	15	24	−0.644	−0.438	21.39 (2.48)	22.22 (2.04)	4.17	<0.001
Social	8	18	−0.112	−0.682	14.24 (2.44)	15.74 (2.03)	6.76	<0.001
Caring Total	78	120	−0.244	−0.507	103.84 (10.30)	108.75 (9.25)	6.84	<0.001

**Table 2:** Results of paired *t*-test in male and female students’ learning performances at pre-test and post-test.

Variables	Gender	Pre-Test (T0)	Post-Test (T1)	*t*	*p*
		Mean (SD)	Mean (SD)		
Assistance	Male	48.13 (4.62)	48.87 (4.03)	−2.31	0.031
	Female	48.44 (4.42)	49.60 (3.88)	−5.16	<0.001
Academic	Male	21.09 (2.31)	21.52 (2.37)	−1.95	0.064
	Female	21.15 (2.34)	21.56 (2.29)	−3.90	<0.001
Affective	Male	21.98 (2.12)	22.22 (1.83)	−1.44	0.164
	Female	21.70 (2.38)	22.22 (2.14)	−3.97	<0.001
Social	Male	15.35 (2.58)	16.30 (2.10)	−4.14	<0.001
	Female	14.78 (2.30)	15.51 (1.97)	−5.33	<0.001
Caring Total	Male	106.54 (10.06)	108.87 (9.31)	−3.48	0.002
	Female	106.08 (10.12)	108.70 (9.31)	−5.90	<0.001

## Data Availability

These study data are de-identified participant data. The data that support the findings of this study are available beginning 12 months and ending 36 months following the article’s publication from the corresponding author, W.-F.M., upon reasonable request at lhdaisy@mail.cmu.edu.tw.
